# *MEIS1* and Restless Legs Syndrome: A Comprehensive Review

**DOI:** 10.3389/fneur.2019.00935

**Published:** 2019-08-28

**Authors:** Faezeh Sarayloo, Patrick A. Dion, Guy A. Rouleau

**Affiliations:** ^1^Department of Human Genetics, McGill University, Montreal, QC, Canada; ^2^Montreal Neurological Institute, McGill University, Montreal, QC, Canada; ^3^Department of Neurology and Neurosurgery, McGill University, Montreal, QC, Canada

**Keywords:** restless legs syndrome, *MEIS1*, iron, sleep disorders, neurogenetics

## Abstract

Restless legs syndrome (RLS) is a common sleep-related disorder for which the underlying biological pathways and genetic determinants are not well understood. The genetic factors so far identified explain less than 10% of the disease heritability. The first successful genome-wide association study (GWAS) of RLS was reported in 2007. This study identified multiple RLS associated risk variants including some within the non-coding regions of *MEIS1*. The *MEIS1* GWAS signals are some of the strongest genetic associations reported for any common disease. MEIS1 belongs to the homeobox containing transcriptional regulatory network (HOX). Work in *C. elegans* showed a link between the *MEIS1* ortholog and iron homeostasis, which is in line with the fact that central nervous system (CNS) iron insufficiency is thought to be a cause of RLS. Zebrafish and mice have been used to study the *MEIS1* gene identifying an RLS-associated-SNP dependent enhancer activity from the highly conserved non-coding regions (HCNR) of *MEIS1*. Furthermore, this gene shows a lower expression of mRNA and protein in blood and thalamus of individuals with the *MEIS1* RLS risk haplotype. Simulating this reduced *MEIS1* expression in mouse models resulted in circadian hyperactivity, a phenotype compatible with RLS. While *MEIS1* shows a strong association with RLS, the protein's function that is directly linked to an RLS biological pathway remains to be discovered. The links to iron and the enhancer activity of the HCNRs of *MEIS1* suggest promising links to RLS pathways, however more in-depth studies on this gene's function are required. One important aspect of *MEIS1*'s role in RLS is the fact that it encodes a homeobox containing transcription factor, which is essential during development. Future studies with more focus on the transcriptional regulatory role of MEIS1 may open novel venues for RLS research.

## Introduction

Restless legs syndrome is a common neurological disorder. The prevalence of RLS cases based on the minimum diagnostic criteria of the international RLS study group (IRLSSG) was estimated between 3.9 and 14.3% of the adult population. It is more common in women than men and the prevalence increases with age in the European and North American populations (1). RLS is more prevalent in people with iron deficiency or kidney disease (2). Twin studies and a familial aggregation analysis estimated the heritability of RLS between 54.0 and 69.4%, thus there is a strong genetic element to the disease (3–5). RLS is a complex condition and environmental factors also contribute to its development.

The first attempts to identify RLS genetic risk factors used genome wide linkage (GWL) approaches in large multiplex families, most of which had an autosomal dominant mode of inheritance and one with autosomal recessive inheritance pattern. The GWL identified loci with large genomic regions, but no causative variant was identified by this approach and the results have often not been reproducible (5–12). Considering the complex nature of RLS, the genetic studies on RLS moved forward to association studies in search for common variants with low to moderate effect size.

## Significance of *MEIS1* in the RLS Genetics Studies

RLS is the first common sleep disorder for which genome wide association studies (GWAS) was performed and genetic risk loci identified. In 2007, the first genome-wide association study on RLS using 401 patients with familial RLS and 1,644 control individuals of German and French-Canadian origin identified common variants in three noncoding genomic regions (13). The strongest association signal found is a 32 kb linkage disequilibrium block in the intron 8 of *MEIS1* gene (13). This association has been replicated in several follow up studies in both familial and sporadic RLS cases (odds ratio 1.92, 95% CI 1.85–1.99, *p*-value = 2.00E−280, from the latest report in 2017) (14–17). Furthermore, common genetic variants with low effect size were identified for RLS in 18 additional loci that each confer a small risk for the disease (17). MEIS1 is a homeobox transcription factor that belongs to the three amino acid loop extension (TALE) family of homeodomain proteins; it is known to have functions in hematopoiesis and vascular patterning (18, 19). This protein forms heterodimeric or heterotrimeric complexes with PBX or HOX proteins for higher DNA binding specificity and affinity (20). It also plays roles in neurodevelopment as well as the development of proximodistal limb axis, with high expression in dopaminergic neurons of substantia nigra and red nucleus (21–24).

Rare coding variants of *MEIS1* were also proposed to contribute to be the cause of RLS. An Arg272-to-His (p.R272H) was found in one of 71 familial probands with RLS. However, a case-control genotyping study of this mutation across a North American cohort failed to validate this variant (25). In another study, an excess of rare null alleles specific to *MEIS1* isoform 1 was observed in RLS cases compared to controls in a burden test on a German population (26). Lastly, in a study conducted by Xiong et al. the thirteen *MEIS1* exons (and their respective splice junctions) were sequenced in 285 familial probands with a confirmed clinical diagnosis and no variants were identified (27). Hence coding variants are at most a very rare cause of RLS. This is not surprising as the gene is involved in many different developmental processes, so functional coding variants would likely have many additional manifestations, in addition to RLS.

## Reduced *MEIS1* Expression may Contribute to the Development of RLS

**Figure 1:**
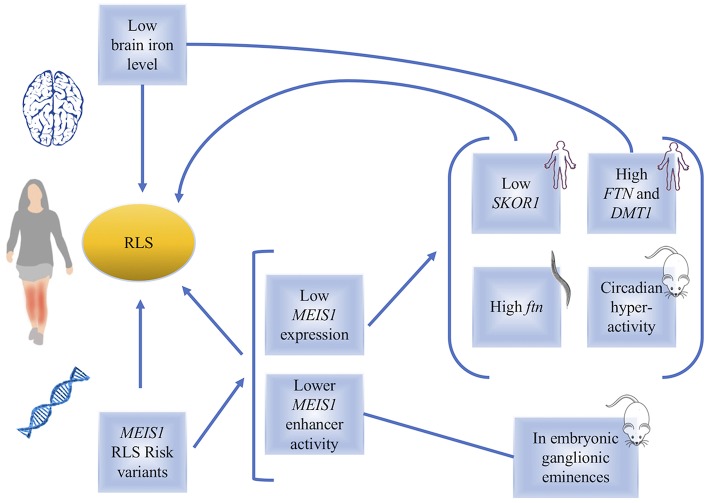
A diagram, summarizing the links between *MEIS1* and restless legs syndrome. Low brain iron level or genetic variations in *MEIS1* have been found in RLS patients. The consequences of low *MEIS1* expression have been identified in human, *C. elegans* and mouse models (indicated in the figure).

After the publication of the first GWAS on RLS, a subsequent study by Xiong et al. (27), used human lymphoblastoid cell lines (LCL) as well as two different brain regions (thalamus and pons) from RLS patients for an expression study. A q-RT-PCR followed by western blot analysis showed that the patients who harbor the *MEIS1* risk haplotype (GG/GG, rs12469063–rs2300478) express lower levels of *MEIS1* mRNA and protein in LCL and thalamus (Figure 1). The authors argued that lower *MEIS1* expression in a subset of individuals can contribute to the development of RLS symptoms (27).

Given the lower *MEIS1* expression in a subgroup of patients, *in vivo* studies on mouse models with heterozygous *Meis1* knockout were conducted (23, 28). Young male and female mice showed hyperactivity with no effect on anxiety-related behaviors, providing a potential animal model to study RLS (Figure 1). Furthermore, considering the age-related manifestation of RLS symptoms in human, effects of *Meis1* haploinsufficiency was also studied in middle aged mice in a study by Salminen et al. (29). *Meis1* haploinsufficiency was associated with a sex dependent increase in the activity more specific to the initial of the rest phase of animal, similar to the circadian rhythm of RLS symptoms observed in human patients. Effects on sensorimotor system in a sex dependent manner were also reported (29).

## The Links Between *MEIS1* and Iron Metabolism

Observations made using a *Caenorhabditis elegans* model showed that post developmental inactivation of *Unc-62* (*MEIS1* ortholog) makes it one of the 64 genes that increase the worm's life span. *Unc-62* in the worms has 25% identity with *MEIS1* and almost 80% identity with its homeodomain. Adding iron chelators in the culture media of the worms resulted in changes in the effect of *Unc-62* inactivation on worms' lifespan, suggesting that *Unc-62* is involved in iron metabolism. The link between *Unc-62* and iron was also measured through its impact on the expression of ferritin. It was found that post developmental inactivation of *Unc-62* using RNAi resulted in higher expression of ferritin, a protein that plays a key role in iron metabolism through its ability to store excess iron and to release it into a soluble and nontoxic form (30). Thalamus samples obtained from RLS patients also showed that individuals with the *MEIS1* risk haplotype, who also have a reduced expression of *MEIS1*, show an increased expression of both ferritin (FTN) light and heavy chains. Furthermore, *DMT1* gene expression was higher in these individuals. *DMT1* is a proton-coupled metal transporter that carries iron from the extracellular domain to the cytoplasm (Figure 1). This finding also supports a link between *MEIS1* and iron metabolism where DMT1 transports iron into the brain (31). More work remains to be done to clarify the exact role of *MEIS1* in iron metabolism relevant to RLS (32). Recent studies suggest that DMT1 is expressed in endosomes of brain capillary endothelial cells denoting the blood-brain barrier (BBB) (33). The best well-established neurobiological abnormality in RLS is reduced brain iron, despite a normal peripheral iron level. The fact that some, but not all RLS patients, respond to intravenous (IV) iron provides an opportunity to interrogate the underlying pathways differing between responders versus non-responders. This led some studies to focus their attention toward the iron uptake at the blood-brain barrier (34). A preliminary analysis of *MEIS1* expression showed elevated *MEIS1* levels in the microvasculature isolated from RLS brain tissue by comparison to the tissues of control individuals. Moreover, a cell culture model of the BBB showed that treatment with an iron chelator increased the *MEIS1* expression, while iron loading conversely decreased *MEIS1* expression (unpublished data presented in a review article on the links between iron and RLS by Connor et al.; the small sample size in this report indicates that more investigations remain to be done to further validate this observation) (35, 36). These data suggest a novel role for *MEIS1* in the BBB that warrants further examination. There are also observations revealing peripheral hypoxia to be associated with RLS symptoms (37). Hypoxia pathway is activated in a number of cell types of RLS patients; this activation can result from or be related to cellular iron deficiency (38). Another study used LCLs and showed *MEIS1* down regulation by RNAi techniques resulted in an increase in transferrin-2 receptor and ferroportin and a decrease in hepcidin mRNA expression (39, 40). The authors suggest that *MEIS1* might control cellular iron transfer to mitochondria and cellular export of iron (40). Putting together all these findings, the data suggest that decreased acquisition of iron by the brain cells is an RLS related pathophysiology, for which a possible role for *MEIS1* can be accounted.

## *MEIS1* has an Allele Dependent Cis-regulatory Function in Telencephalon (a Study in Mice and Zebrafish)

A cluster of highly conserved non-coding regions (HCNRs) in the *MEIS1* locus suggests the presence of cis-regulatory elements (23). Considering that most variants found by GWAS are located in the regulatory regions, Spieler et al. conducted a study to identify the cis-regulatory role of the common intronic variants in *MEIS1* HCNRs (23). They studied an RLS associated variant (rs12469063), which is in the HCNR 617 of *MEIS1*, in transgenic mice and zebrafish using a reporter assay. They found that in mice, rs12469063 lies within a region of high interspecies conservation with neural enhancer activity and has an allele-specific functional impact. This study found that the risk allele of rs12469063 decreases the enhancer activity of this region in LGE and MGE (lateral and medial ganglionic eminences). The effect of rs12469063 on *MEIS1* enhancer function in the LGE/MGE region suggests that RLS may involve the basal ganglia because these regions give rise to the basal ganglia [also discussed in a review by Salminen et al. (41)]. The enhancer activity of HCNR harboring the RLS associated rs12469063 happens in this mouse model during development, which suggests its predisposition to RLS occurs during embryonic development (Figure 1). Affinity chromatography showed that CREB1 has higher binding affinity to the RLS risk allele compared to the protective allele of rs12469063. A reporter screen in the zebrafish confirms the enhancer activity of HCNR observed in the mice and found two more transcriptionally active enhancers to this region. HCNR 617 harboring rs12469063 is the only RLS-SNP dependent enhancer region (23, 42).

## MEIS1 Regulates *SKOR1*

Expression studies of the RLS associated loci of *BTBD9, MAP2K5*, and *SKOR1* (previously called *LBXCOR1*) did not find changes in their levels of expression in lymphoblasts or two brain regions (pons and thalamus) of RLS patients (43). However, RLS patients with the *MEIS1* risk haplotype were observed to have a reduced expression of *SKOR1*, in addition to a reduced expression of *MEIS1* (Figure 1) (43). Follow up studies using siRNA targeting *MEIS1*, electromobility shift assays and luciferase reporter assays suggested the expression of *SKOR1* to be under the regulatory control of MEIS1. This transcription factor action of MEIS1 is due to its direct binding on two distinct promoter regions of *SKOR1* (43). Hence the dysregulation of MEIS1 might predispose to RLS both directly and indirectly, possibly throughout its regulatory role on other genes like *SKOR1*. A new SNP reported in this study is 8.7 kb upstream *SKOR1* ATG start site, which acts as a regulatory SNP (rSNP). The risk allele in this locus reduces the binding affinity of MEIS1 to the *SKOR1* promotor and results in reduced expression of *SKOR1* (43). SKOR1 acts as the transcriptional corepressor of LBX1, a homeodomain transcription factor. Skor1 expression in the Mice embryonic CNS is present in a certain subset of post-mitotic neurons generated posterior to the midbrain-hindbrain border. Skor1 is selectively expressed in the dorsal horn interneurons of developing spinal cord in Mice, where Lbx1 is required for proper specification. It is suggested that SKOR1 probably mediates the sensory inputs of RLS, among others. Despite the importance of SKOR1 in RLS genetic, only little is known regarding the actual function of this gene in RLS underlying pathways. The current literature only suggests that MEIS1 dysregulation may causes SKOR1 dysregulation possibly leading to the sensory phenotypes of RLS (44).

## *MEIS1* and Other Sleep Related Disorders Like Insomnia, PLMS and RBD

Insomnia is characterized by problems in falling asleep or maintaining asleep. With a heritability estimate of 38 and 59% in men and women, respectively, genetic factors must play a crucial role in insomnia. Recent genetic studies of insomnia using cases from the UK biobank showed that *MEIS1* has the strongest association signal, suggesting *MEIS1* may be a shared genetic risk factor for RLS and insomnia (45–47). Some reports argue that the phenotype overlap could only drive some, and not all of the *MEIS1*'s association with insomnia, thus suggesting that *MEIS1* has a pleiotropic effect on RLS and insomnia (45). However, other reports suggest that the association of *MEIS1* with insomnia only comes from the inclusion of RLS cases (48). Such inconsistencies might be due to the heterogeneous phenotypic definition of insomnia itself which can lead to the inclusion of a substantial number of RLS cases. Furthermore, GWAS on periodic leg movement during sleep (PLMS), which are present in approximately 80% of RLS cases, also shows association with *MEIS1* (49–53). This pleiotropic effect can arise from *MEIS1*'s wide expression pattern during the development (54). So far, no genetic links between RBD (REM sleep behavior disorder) and *MEIS1* have been reported.

## Conclusion

MEIS1 region is one of the several loci found to be associated with RLS genetic, which overall explain less than 10% of RLS heritability. The many roles for *MEIS1* in development make the study of its role in RLS challenging. MEIS1 establishes motor neuron pool identity and their target-muscle connectivity (55), it also regulates the proximodistal limb axis development (21). This protein is highly expressed in dopaminergic neurons of the substantia nigra and red nucleus, though what it does in these cells remains unknown (22–24). Meanwhile, the biology of RLS is poorly understood, with the most consistent abnormality being altered iron homeostasis with brain iron deficiency (56–59). Data presented in this report suggest that the role of *MEIS1* in RLS involves, among possibly other functions, altered iron homeostasis via altered transcriptional regulatory activity in RLS pathways. More in depth follow up studies on the function of MEIS1 with more focus on its regulatory role as a transcription factor might shed more light on the underlying pathways involved in RLS. To reach this goal, it will be essential to have access to RLS patients brain material carrying different genotypes of the RLS GWAS signals and to detailed clinical data to be used as covariates in the analyses. This combination would increase the likelihood of identifying elements that are critical to the onset and progression of RLS. This clinical data includes patient iron levels, their response to medication, age at onset, familial or sporadic RLS, presence or absence of PLMS, diagnosis by a physician and information about the patients' other health conditions. The next step could involve the use of model organisms to further validate and investigate RLS related mechanisms.

## Author Contributions

FS contributed to the original concept of the article, did the literature search, wrote the original version of the manuscript and reviewed it as it progressed. PD and GR contributed to the original concept of the manuscript and reviewed the manuscript.

### Conflict of Interest Statement

The authors declare that the research was conducted in the absence of any commercial or financial relationships that could be construed as a potential conflict of interest.
